# Conformational Changes in Two Inter-Helical Loops of Mhp1 Membrane Transporter

**DOI:** 10.1371/journal.pone.0133388

**Published:** 2015-07-17

**Authors:** Hyun Deok Song, Fangqiang Zhu

**Affiliations:** Department of Physics, Indiana University—Purdue University Indianapolis, Indianapolis, Indiana, United States of America; University of Minnesota, UNITED STATES

## Abstract

Mhp1 is a bacterial secondary transporter with high-resolution crystal structures available for both the outward- and inward-facing conformations. Through molecular dynamics simulations of the ligand-free Mhp1 as well as analysis of its crystal structures, here we show that two inter-helical loops, respectively located at the extra- and intracellular ends of the “hash motif” in the protein, play important roles in the conformational transition. In the outward- and inward-facing states of the protein, the loops adopt different secondary structures, either wrapped to the end of an alpha-helix, or unwrapped to extended conformations. In equilibrium simulations of 100 ns with Mhp1 in explicit lipids and water, the loop conformations remain largely stable. In targeted molecular dynamics simulations with the protein structure driven from one state to the other, the loops exhibit resistance and only undergo abrupt changes when other parts of the protein already approach the target conformation. Free energy calculations on the isolated loops further confirm that the wrapping/unwrapping transitions are associated with substantial energetic barriers, and consist of multiple sequential steps involving the rotation of certain backbone torsion angles. Furthermore, in simulations with the loops driven from one state to the other, a large part of the protein follows the loops to the target conformation. Taken together, our simulations suggest that changes of the loop secondary structures would be among the slow degrees of freedom in the conformational transition of the entire protein. Incorporation of detailed loop structures into the reaction coordinate, therefore, should improve the convergence and relevance of the resulting conformational free energy.

## Introduction

Secondary transporters [1,2] are active membrane transporters that simultaneously transfer two or more solute species across the membrane. Without directly consuming energy sources such as light or ATP, secondary transporters can utilize the gradient of one solute to pump the other against its concentration, thus achieving active transport. Secondary transporters consist of symporters (or co-transporters) and antiporters (or exchangers), which transfer the two solutes in the same or the opposite direction, respectively. Despite the vast variety of membrane transporters, they are believed to exploit a common molecular mechanism—the alternating access model [3,4], which was proposed long before any transporter structure was known. According to this model, membrane transporters may adopt an outward-facing (OF) or an inward-facing (IF) conformation, with the interior of the protein accessible to the extra- or intracellular solution, respectively. The alternating transitions between these two conformations are then coupled to the translocation of the solutes [3,4].

In the past decade, a rapidly growing number of crystal structures have been determined for secondary transporters. Despite low sequence similarity, some of the transporters share a common structural fold, the LeuT fold [1], in which the two halves of the protein, each with five transmembrane (TM) helices, exhibit an inverted repeat topology and are related by a pseudo two-fold internal symmetry [5,6]. Several members [7–18] in the LeuT class are Na^+^-coupled secondary active transporters [19,20], which may utilize the Na^+^ electrochemical gradient to co-transport the substrates into the cell. Based on the crystal structures, a large body of computational studies [21–32], particularly molecular dynamics (MD) simulations, has been carried out to further investigate the dynamics of the LeuT-fold Na^+^-coupled transporters.

The bacterial hydantoin transporter Mhp1 is one of few membrane transporters with high-resolution crystal structures available for both the OF and IF conformations, and thus an excellent system to study the alternating access model in atomic details. The ligand-free OF and IF structures, as well as several OF structures with bound Na^+^ ion and substrates/inhibitors, have been solved for Mhp1 by X-ray crystallography [14–16]. Mhp1 adopts the LeuT fold, and co-transports Na^+^ ion and hydantoin (or derivatives such as benzyl-hydantoin) into bacterial cells. In addition to crystallography, Mhp1 has also been studied by biochemical assays [16,33] and spin-labeled electron paramagnetic resonance measurement [34].

Several successful computational studies have been performed on the Mhp1 transporter. Beckstein et al. [15] applied dynamic importance sampling as well as unbiased MD simulations to examine a proposed transition between the OF and IF conformations, and recently used MD [16] to reveal the interactions between Mhp1 and various ligands. Based on coarse-grained protein models, Adelman et al. employed weighted ensemble path-sampling to identify the transition pathways for Mhp1 [29]. Li et al. performed long unbiased MD simulations which revealed reversible formation of water chains in Mhp1 and several other membrane transporters [30]. In a remarkable achievement, Zhao and Noskov adopted a variety of simulation methods and reported the thermodynamics for the entire conformational cycle of Mhp1 [31]. Recently, Islam and Roux used MD simulations to calculate the distance distributions between the experimentally introduced spin labels in Mhp1 [34] and the homologous LeuT transporter [35]. In the conformational studies of Mhp1 and other membrane transporters, free energies are typically defined by some holistic reaction (progress) coordinates such as the distance-RMSD [29] or the distances [15,31] and relative orientations [36] between protein domains.

**Fig 1:**
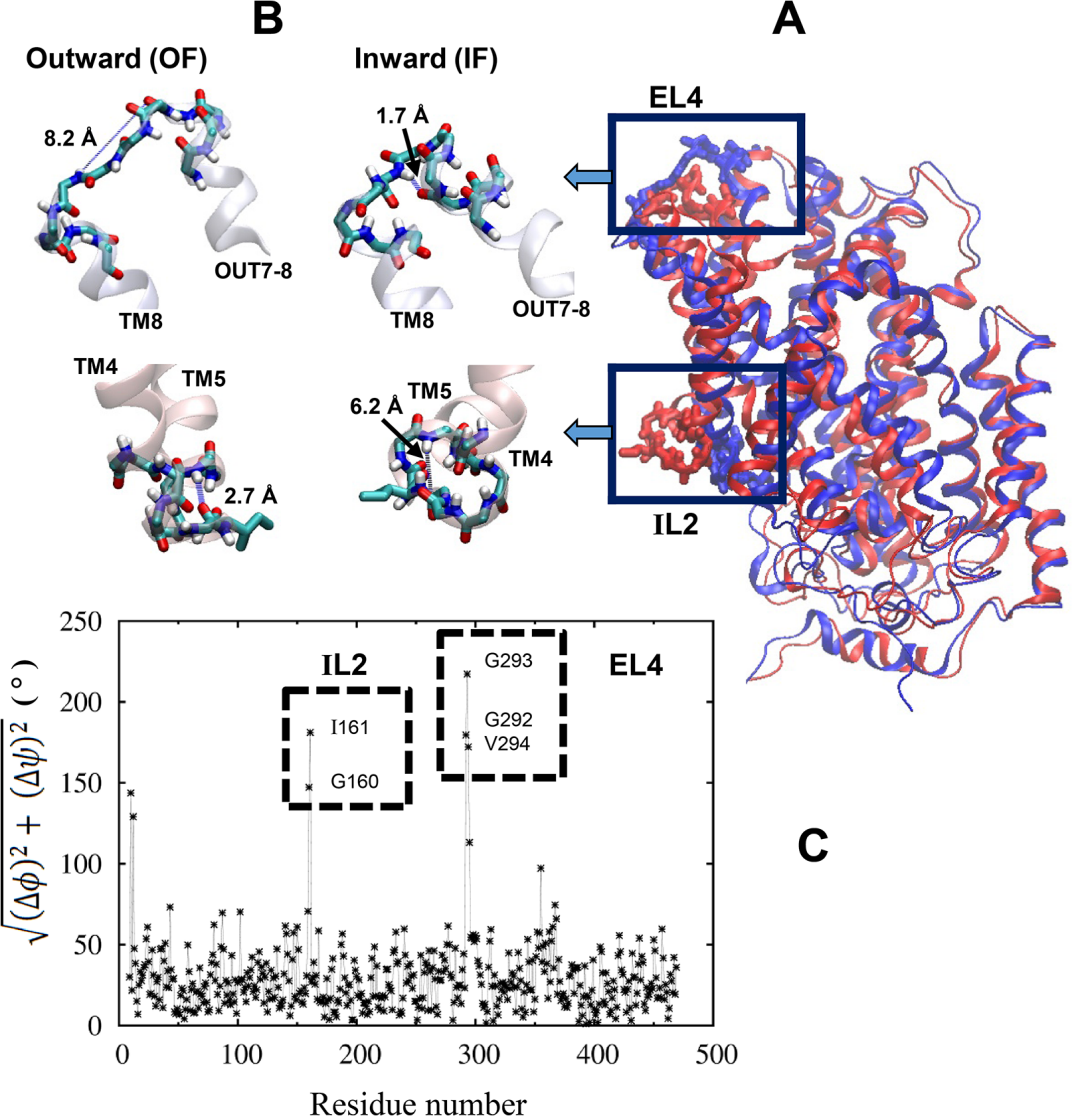
Comparison of the OF [14] and IF [15] crystal structures of Mhp1. **(A)** A superimposition of the two crystal structures, with the extracellular side up. The EL4 extracellular loop (between the OUT7-8 helix and TM8) and the IL2 intracellular loop (between TM4 and TM5) are highlighted. **(B)** The backbone structures for the EL4 and IL2 loops in the OF and IF conformations. The side chain of Ile161 in the IL2 loop is also shown. Two distances, between S295:H and V291:O in the EL4 loop and between I164:H and G160:O in the IL2 loop, are labeled. **(C)** Difference in the backbone torsions. For each residue, the differences in the backbone φ and ѱ angles between the OF and IF crystal structures are calculated, and the combined deviation $\sqrt{{\left(\Delta \varphi \right)}^{2}+{\left(\Delta \psi \right)}^{2}}$ is plotted.

In our preliminary attempt to identify a transition pathway between the OF and IF conformations, we noticed discontinuity in some local secondary structures in Mhp1. In fact, the significant difference is directly evident in the OF [14] and IF [15] crystal structures (Fig 1). A comparison of the backbone φ and ѱ torsion angles for each residue, as shown in Fig 1C, identifies two short loops with high deviations between the two Mhp1 structures: one loop (termed EL4 here) connecting a short extracellular helix (OUT7-8) to TM8, and the other (termed IL2) between TM4 and TM5 on the intracellular side. The large differences in the backbone torsions indicate distinct secondary structures, as visualized in Fig 1B. In the EL4 loop, Ser295 is wrapped to the C-terminal end of the OUT7-8 helix in the IF state and could readily form a canonical α-helical H-bond with Val291. In the OF state, in contrast, this last turn of the OUT7-8 helix is completely unwrapped, such that Val291 becomes the end of the helix, with the original H-bond partners (the amide hydrogen in Ser295 and the carbonyl oxygen in Val291) now separated by 8.2 Å. For the IL2 loop, in the OF state, Gly160 is wrapped to the N-terminal end of TM5 and within reasonable H-bond distance to Ile164. But this turn of the α-helix is unwrapped in the IF state, such that the H-bond partners from Gly160 and Ile164 are now 6.2 Å apart (Fig 1B). As a result of the different backbone conformations, the side chain of Ile161 in this loop adopts almost opposite orientations in the two crystal structures, pointing toward the protein interior in the OF state and away from the protein in the IF state (Fig 1B). The loop geometries in the available ligand-bound crystal structures for Mhp1 [14,16] are highly similar to the OF state here.

In our preliminary sampling of Mhp1 conformation along reaction coordinates based on the C_α_ atoms, the backbone torsions of the EL4 and IL2 loops did not follow the reaction coordinate. For example, when moving restraints on the C_α_ atoms were applied to drive Mhp1 from the OF to the IF state following a predefined pathway, some backbone φ and ѱ angles in the two loops remained highly different from those in the IF structure even when the C_α_ positions (and other order parameters such as inter-domain distances) reached the IF state. Consequently, the apparent free energy of the IF state (with incorrect secondary structures for the loops) appeared to be much higher than the OF state. Reversely, when the C_α_ atoms were driven from the IF to the OF state, the two loops also did not approach the correct secondary structures in the OF conformation, again resulting in a high free energy for the target state. The slow equilibration of the loop structures therefore gave rise to significant hysteresis in the calculation of conformational free energy.

Given that the EL4 and IL2 loops may contribute to the slow degrees of freedom in the conformational transition of Mhp1, it would be helpful to better understand how they change their structures. In this focused study of the two loops, we aim to identify and evaluate plausible transition pathways between the OF and IF conformations of the loops. We also assess the significance of the loops in the conformational change of the entire protein.

## Methods

Ideally, unbiased MD simulations without any artificial restraints are the most reliable computational tool to observe equilibrium behaviors of the system. However, major conformational transitions in large proteins occur on time scales much longer than what typical MD simulations can currently afford. Therefore, the usage of the unbiased simulations is normally limited to examining the stability of a given protein structure as well as small-scale structural relaxations near the initial state, rather than revealing spontaneous major conformational transitions. In contrast, biased simulation approaches could enforce large conformational changes. In steered molecular dynamics (SMD) simulations [37], e.g., a moving harmonic restraint is applied on a chosen reaction coordinate (such as a distance between atoms or protein motifs) to drive the system from one state to the other. Targeted molecular dynamics (TMD), as recently applied on Mhp1 [31], is essentially one type of SMD, in which the root mean square distance (RMSD) to a target structure is chosen as the reaction coordinate. TMD could be a natural choice to produce a conformational transition in the simulation when the detailed transition pathway between the two end structures is not known. In SMD and TMD, the system is in a nonequilibrium state and typically exhibits hysteresis due to the fast-moving driving restraints. Enhanced sampling techniques, such as the string method [38,39], can be applied to relax the conformations along a given transition pathway and to obtain a free energy profile that describes the reversible transition.

We adopted a combination of the MD techniques above to explore the conformational changes in the ligand-free Mhp1, with a special focus on the EL4 and IL2 loops. In this section, we describe the details of these simulations.

### System Setup and Equilibration

**Fig 2:**
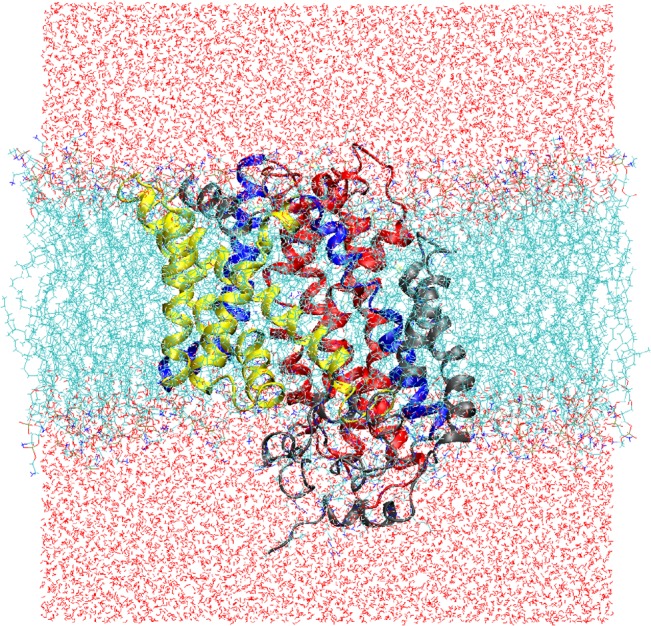
Simulation system. The protein Mhp1 (in the IF conformation) is embedded in a POPE lipid bilayer.

We constructed two simulation systems with Mhp1 initially in the OF (PDB: 2JLN [14]) and IF (PDB: 2X79 [15]) conformations, respectively. The protein in both systems consists of residues 6 to 470. The bound ions in the crystal structure [14] were removed. We adopted the standard protonation states at pH 7 for all residues. In particular, all His residues are neutral, with the proton at the ε position. For both systems, to embed the protein in a lipid bilayer, we first manually positioned and oriented the protein to match its hydrophobic surface to the membrane interior. Then the *membrane* plugin in the VMD software [40] was used to combine the protein with a 1-palmitoyl-2-oleoyl-*sn*-glycero-3-phosphoethanolamine (POPE) lipid bilayer. POPE is the major lipid component of bacterial membranes, and was also used in other MD simulations of Mhp1 [15,31]. The system consists of 200 POPE lipids after removing those overlapping with the protein. The system was then solvated by 15,957 water molecules, and four Cl^-^ ions were added to render it electrically neutral. In both cases the simulation system (Fig 2) contains a total of 80,145 atoms.

**Fig 3:**
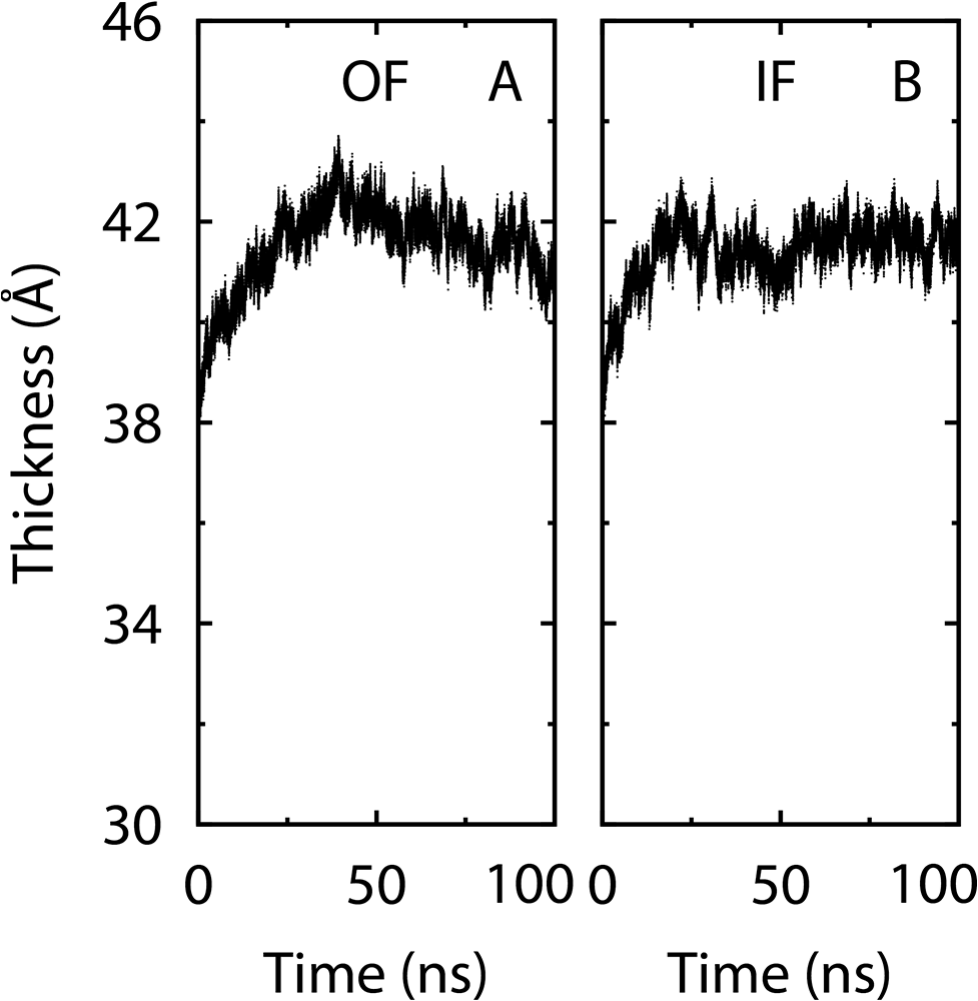
Thickness of the lipid bilayer. The thickness was calculated as the difference between the average *z* coordinates (membrane plane normal to the *z* axis) of the lipid phosphorus atoms in each leaflet. (**A**) Results for the unbiased simulation with Mhp1 in the OF state. (**B**) Results for the IF-state simulation.

For each system, we first performed a short equilibration for the lipids, water and ions. Specifically, we ran a conjugate-gradient minimization of 2,000 steps in which all protein coordinates were fixed. With the protein still fixed, we then performed an equilibrium simulation of 2 ns under the NPT ensemble (with details described further below), in which the temperature of the system was raised to and maintained at 300 K by Langevin dynamics. Next, we relaxed the protein and similarly performed another minimization and an equilibrium simulation of 20 ns (also under NPT), in which no atom was fixed or restrained. The average orientation of the protein from this free equilibrium simulation was used as the reference in some restrained simulations described later. We later extended the two unbiased equilibrium simulations to 100 ns. The thickness (Fig 3) of the lipid bilayer appeared to be stabilized in these simulations.

All simulations were performed using the CHARMM (Ver. c36) force field for the protein (with the CMAP correction) [41–43] and lipids [44], the TIP3P water model [45], and the NAMD2 (Ver. 2.9) program [46], with a time step of 2 fs. All bond lengths involving hydrogen atoms were constrained using the SHAKE [47] and SETTLE [48] algorithms. We adopted a cutoff distance of 12 Å for nonbonded interactions, with a smooth switching function taking effect at 10 Å. Full electrostatics was calculated every 4 fs using the particle-mesh Ewald method [49]. Temperature was maintained at 300 K by Langevin dynamics with a damping coefficient of 0.1 ps^-1^. All simulations for the protein in explicit lipids and solvent were under the periodic boundary conditions and a constant pressure of 1 atm achieved using the Nose-Hoover Langevin piston method [50], with the size of the periodic box allowed to fluctuate but the lengths in *x* and *y* kept equal to each other. In equilibrated systems, the periodic box has dimensions of ~84 Å × ~84 Å × ~110 Å.

### Targeted Molecular Dynamics Simulations

In parallel to the unbiased simulations, we performed two TMD simulations (each 50 ns, NPT ensemble) to enforce transitions between the OF and IF conformations. The TMD simulations apply a harmonic restraint on the RMSD with respect to a target structure:

$$U(\vec{R})=\frac{1}{2}k{\left[\text{RMS}(\vec{R})-r_{0}\right]}^{2},$$

in which $\vec{R}$ is the coordinates of the selected atoms, $\text{RMS}(\vec{R})$ is their RMSD to the target structure, and *r*_0_ is the reference value for the restraint. Here the RMSD restraint was applied to all heavy atoms of the protein. In our first TMD simulation, referred to as TMD_O→I_, the initial coordinates were the same as those at the beginning of the unbiased simulation for the OF state, when the protein was fixed but other parts of the system had been equilibrated for 2 ns, as described earlier. Therefore this initial protein conformation is exactly the same as the OF crystal structure. In the meantime, the IF crystal structure was used as the target in this TMD_O→I_ simulation, with the reference value *r*_0_ decreasing linearly from the initial RMSD of 3.6 Å to 0 in the 50 ns. Our second TMD simulation, TMD_I→O_, was similarly performed for the reverse direction, using the IF crystal structure as the initial coordinates and the OF crystal structure as the target.

### Simulations of Isolated Loops

The purpose of the simulations on each isolated loop is to relax the strain in the loop during the TMD simulations and to obtain a plausible transition pathway between the two conformations of the loop. In these simulations, the system contains only a short segment of the protein that encloses the loop of interest. Specifically, the isolated system for loop EL4 consists of residues 289 to 299, and the system for IL2 consists of residues 155 to 165. No water molecule, lipid or ion was included in these systems, and the simulations were performed with the generalized Born implicit solvent.

The string method [38,39] was used to relax the loop conformations and to refine the transition pathway. In these simulations, the conformation of the loop was represented by some backbone atoms, including the hydrogen atoms (except for proline residues) in the NH groups and the oxygen atoms in the CO groups, as well as all C_α_ atoms. The Cartesian coordinates of these chosen atoms then specify the backbone conformation, including both the position and the orientation of each peptide plane. For the IL2 simulations, we also incorporated the C_β_ atom of the Ile161 side chain into the representative atoms. Starting from the initial transition pathways obtained from the TMD simulations, we performed iterative pathway refinement as similarly done in a previous study [51]. Specifically, to refine a given transition pathway, we first take some evenly spaced points (images) on the pathway curve, each corresponding to an intermediate conformation between the two end structures. All images are aligned with a common reference conformation to eliminate the overall rigid-body translation and rotation of the system. We then perform a group of simulations, each confined to a different image by harmonic restraints on the representative atoms. Hamiltonian replica exchange [52] is implemented to facilitate convergence in these string-method simulations, allowing the swap of two simulations that sample adjacent images. From the simulation trajectories, we calculate the average positions of the representative atoms for each image, and then perform curve fitting [51,53,54] through these average conformations, with the two ends of the curve fixed at the OF and IF conformations in the crystal structures. The fitted curve then represents a new and improved pathway, which can be further refined in a new round of such simulations. A free energy profile along the pathway can be obtained by integrating the mean forces on the images [51].

The initial coordinates in the first iteration of the string-method simulations were taken from the TMD simulations. Specifically, after the images on the initial pathway were determined, the loop snapshot in the TMD trajectory with the smallest RMSD to a given image was taken as the initial coordinates for the simulation at that image. We performed two groups of string-method simulations in parallel, starting from the snapshots in the TMD_O→I_ and TMD_I→O_ simulations, respectively. The two groups of simulations thus started with different initial coordinates and were carried out independently in each iteration, but sampling a common transition pathway and subject to the same set of restraints. After an iteration, the average conformations at the images were calculated from the trajectories of both simulations, and were then used to determine (through curve fitting) the new common pathway for both groups of the simulations in the next iteration. A comparison of the two simulations with the same restraining potential but different initial coordinates thus offers an evaluation for the convergence of the sampling.

During the pathway refinement, some intermediate metastable states emerged as local minima in the free energy profile. The free energy minima divide the entire pathway into multiple sections, each corresponding to a partial transition from one intermediate (or terminal) state to another. When updating the pathway, instead of fitting a single curve for the entire transition, we fit a smooth curve for each partial transition between major intermediate states. The piecewise curve fitting here ensures that each individual partial transition is smooth while allowing large changes in the curve direction at the intermediate states.

In this study, we performed a few iterations of string-method simulations for the pathway refinement, using 60 images (including the two ends) on the pathway and a spring constant of 2 kcal/mol/Å^2^ for the harmonic restraint on each representative atom. On the final pathway, we carried out a set of longer simulations (20 ns per image) with 120 images and 4 kcal/mol/Å^2^ for the spring constant, to obtain a more accurate estimation of the free energy along the transition.

### Loop-driven Simulations

To examine the coupling between the loops and other parts of the protein, we carried out simulations (NPT ensemble) in which only the atoms in the EL4 and IL2 loops were subject to restraints moving from one state (OF or IF) to the other. Because the loop conformations are represented by the Cartesian coordinates, we applied strong harmonic restraints [54] on the center (with spring constant 1,000 kcal/mol/Å^2^) and the orientation angle (200 kcal/mol/degree^2^) of the entire protein to eliminate the overall rigid-body translation and rotation. The reference for the orientation restraint has the conformation in the OF crystal structure and the average orientation (tilt) angle from the unbiased equilibrium simulations described earlier. With these restraints, the center and orientation of the protein remained essentially constant in the loop-driven simulations, such that the Cartesian coordinates could unambiguously represent the internal protein conformation, such as the status of the loops with respect to other parts of the protein. We note that, alternatively, it is also possible to apply restraints that are invariant to rigid-body translation and rotation [55,56].

Between the aligned OF and IF crystal structures, the loops differ in their center and orientation as well as their internal conformation. Transition of the loops from one state to the other, therefore, involves evolution of the translational, rotational, and internal degrees of freedom. In our prescribed loop movement here, the evolution of internal loop conformation follows the refined transition pathway from the string-method simulations of the isolated loop. In the meantime, the loop center evolves linearly with the progression parameter from its position in one crystal structure to the other. The evolution of loop rotation is similarly determined by a linear interpolation between the orientation quaternions [57] in the OF and IF states. Both the EL4 and the IL2 loops follow the linear evolution described above. The resulting driving pathways for the loops thus describe a smooth transition of the loop coordinates between the two crystal structures. A total of 60 evenly spaced frames of loop coordinates from this pathway were taken as the references for the moving restraints. We performed two loop-driven simulations, one starting from the OF crystal structure and with the two loops driven to the IF coordinates, and the other in the opposite direction. The initial coordinates in these simulations were the same as those in the TMD simulations. A total of 120 ns were run for each simulation, with each frame of restraints (with a spring constant of 2 kcal/mol/Å^2^) applied for 2 ns on the loop atoms. By advancing the restraint frames successively, the loop coordinates were then gradually driven from one state to the other.

## Results

**Fig 4:**
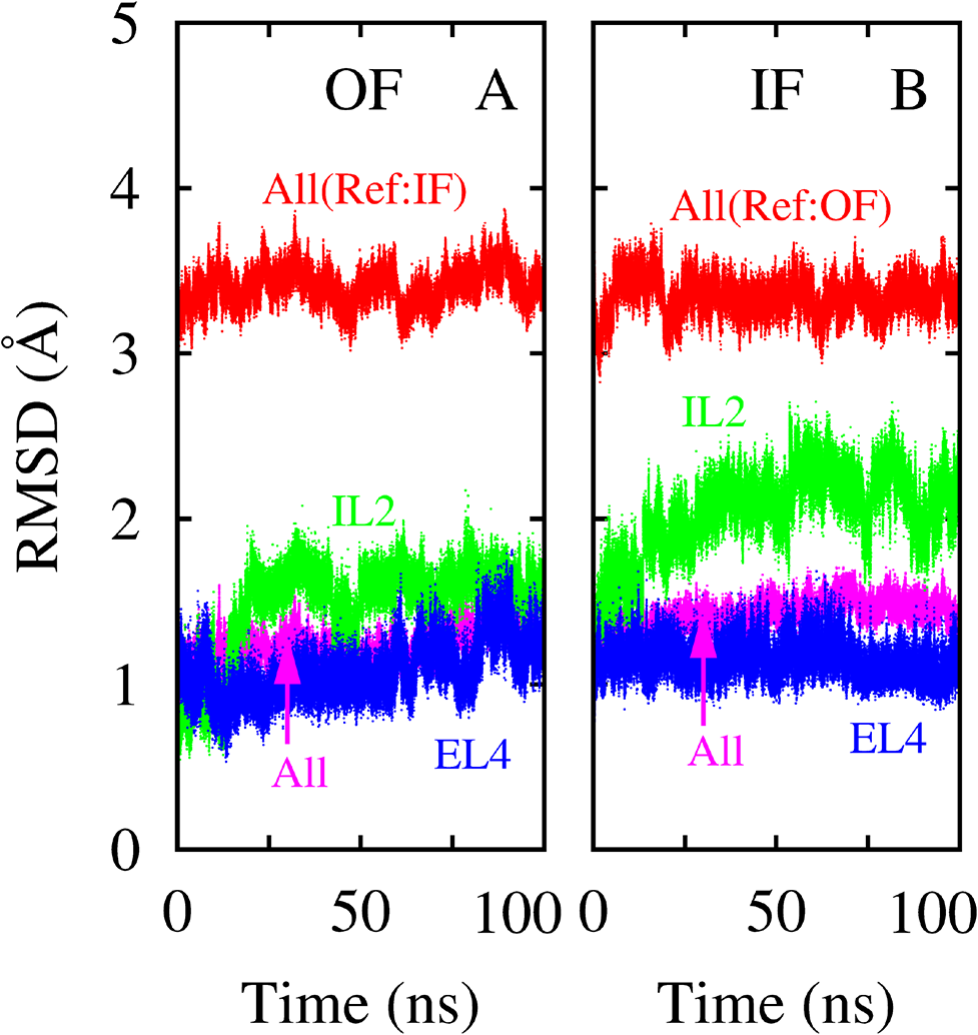
RMSDs from the two unbiased equilibrium simulations. For each simulation, the RMSDs for all C_α_ atoms with respect to the OF and IF crystal structures, as well as the RMSDs for the heavy atoms in the EL4 and IL2 loops with respect to the initial crystal structure, are shown in the figure. (**A**) Results for the simulation with Mhp1 in the OF state. (**B**) Results for the IF-state simulation.

As discussed in Introduction, the EL4 and IL2 loops adopt highly different conformations in the OF and IF crystal structures, manifested by several backbone torsion angles (Fig 1). Here we first examine the stability of the loop conformations in two 100-ns equilibrium simulations starting from the OF and IF crystal structures, respectively. The RMSDs (Fig 4) indicate that the IL2 loop exhibited larger deviations and fluctuations than the EL4 loop, especially in the IF-state simulation. Nevertheless, the overall conformations of the loops as well as the entire protein remained stable, and no major transition toward the opposite state occurred within the 100 ns in either simulation.

We also calculated the dynamic cross-correlation coefficients between the EL4 and IL2 loops defined as

$$C_{ij}\equiv \frac{\left\langle \left({\vec{r}}_{i}-\left\langle {\vec{r}}_{i}\right\rangle \right)\cdot \left({\vec{r}}_{j}-\left\langle {\vec{r}}_{j}\right\rangle \right)\right\rangle }{\sqrt{\left\langle {\left({\vec{r}}_{i}-\left\langle {\vec{r}}_{i}\right\rangle \right)}^{2}\right\rangle \left\langle {\left({\vec{r}}_{j}-\left\langle {\vec{r}}_{j}\right\rangle \right)}^{2}\right\rangle }},$$

in which residues *i* and *j* are from EL4 and IL2, respectively. The averages in the equilibrium trajectory were taken for the expectation values in the equation above. All correlation coefficients calculated from the two simulations are between -0.24 and 0.05, indicating that the two loops did not exhibit very strong correlation in these equilibrium simulations. However, we note that each simulation only sampled a small conformational space near the respective OF/IF crystal structure in the 100 ns. If the simulation were extremely long such that spontaneous transitions between the two states could be sampled, the correlation should be much stronger than the estimates from our simulations here.

To induce the conformational changes, we resorted to biased simulations with applied driving restraints. However, the crystal structures alone did not provide sufficient clues for the transition pathway, especially because some backbone torsions (φ and ѱ) in the loops differ by almost 180° between the OF and IF states, as discussed earlier. In such cases either a counterclockwise or a clockwise rotation of the torsion angle, each corresponding to a qualitatively different transition, would appear equally likely. With multiple torsions involved, therefore, the transition of the loop could in principle follow a combinatorial number of alternative pathways. Instead of exhaustively examining all potential pathways, here we employed TMD simulations to reveal plausible transitions.

### Targeted Molecular Dynamics Simulations

**Fig 5:**
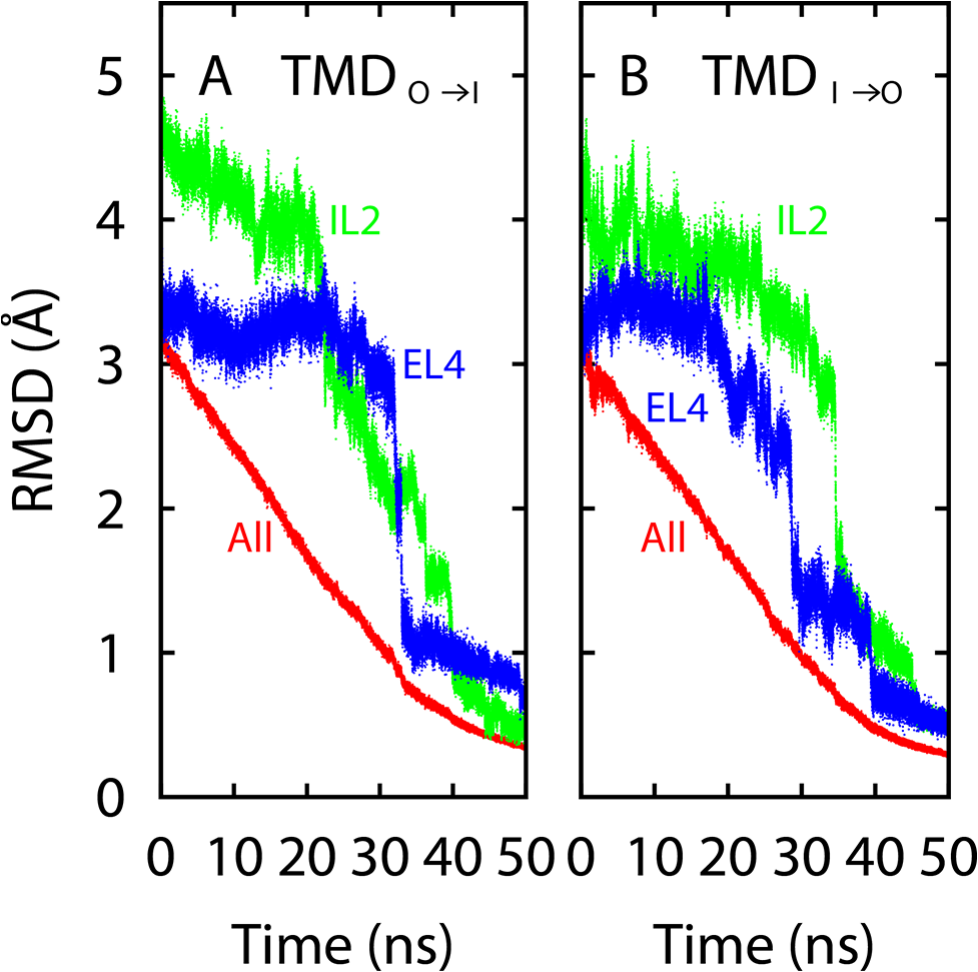
RMSDs from the two TMD simulations. For each simulation, the heavy-atom RMSDs for the EL4 and IL2 loops and the entire protein with respect to the target crystal structure are shown. (**A**) Results for the TMD_O→I_ simulation. (**B**) Results for the TMD_I→O_ simulation.

In TMD, we used the RMSD to the target conformation as the order parameter, and applied moving restraints on this parameter to drive the conformation of the entire Mhp1 toward the target. In the end of the TMD simulations, the conformation of the protein (including the loops) was indeed close to the target crystal structure. Therefore each of the two simulations (TMD_O→I_ and TMD_I→O_) provided a transition between the OF and IF states. In these simulations, the EL4 and IL2 loops were not treated differently, as the RMSD is defined by all heavy atoms of the protein. However, in both TMD_O→I_ and TMD_I→O_, major changes in the two loops occurred in later stages of the simulations, when the overall protein conformation already approached the target structure, as can be seen from the evolution of the loop and all-protein RMSDs (Fig 5). Moreover, the loop RMSDs to the target did not decrease uniformly with time, but instead underwent some abrupt drops during the TMD simulations (Fig 5). These observations imply that the internal conformations of the loops could not rapidly adapt to the conformational change of the entire protein, and that the transitions in the loop conformations would involve nontrivial energetic barriers.

Although induced conformational changes were achieved in the TMD simulations, the protein structure necessarily experienced significant strain due to the fast pulling speed. Furthermore, the two transitions in TMD_O→I_ and TMD_I→O_ are not exactly the reverse of each other. In particular, major conformational changes in the two loops occurred when the overall protein structure was close to the IF and OF states in the TMD_O→I_ and TMD_I→O_ simulations, respectively, a manifestation of hysteresis. Accordingly, we focused on the two loops here and attempted to obtain a reversible transition pathway for each loop when isolated from the protein, as described below.

### Conformational Transitions in the Isolated Loops

**Fig 6:**
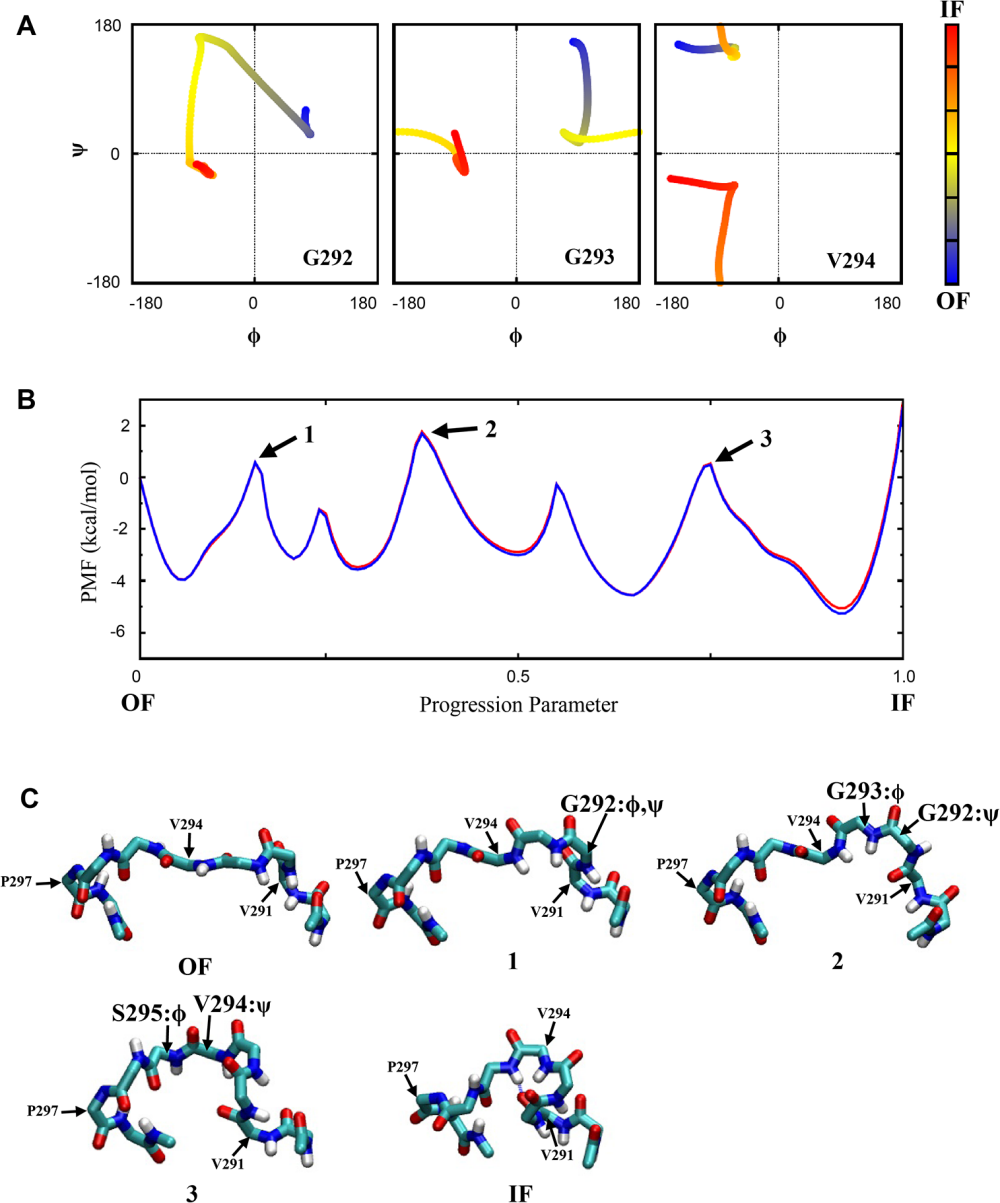
Conformational transition in the isolated EL4 loop. **(A)** The φ and ѱ angles for residues Gly292, Gly293 and Val294 along the transition pathway, with the color representing the progression of the transition, from *blue* for the OF state to *red* for the IF state. **(B)** The calculated free energy profile for the transition. The *blue* and *red* curves (which are largely overlapping) were obtained from two independent groups of string-method simulations with different initial coordinates. **(C)** Some snapshots along the transition pathway between the OF and the IF states, including the backbone structures at the major energetic barriers identified in (B). Major torsion angles involved in each barrier are indicated.

**Fig 7:**
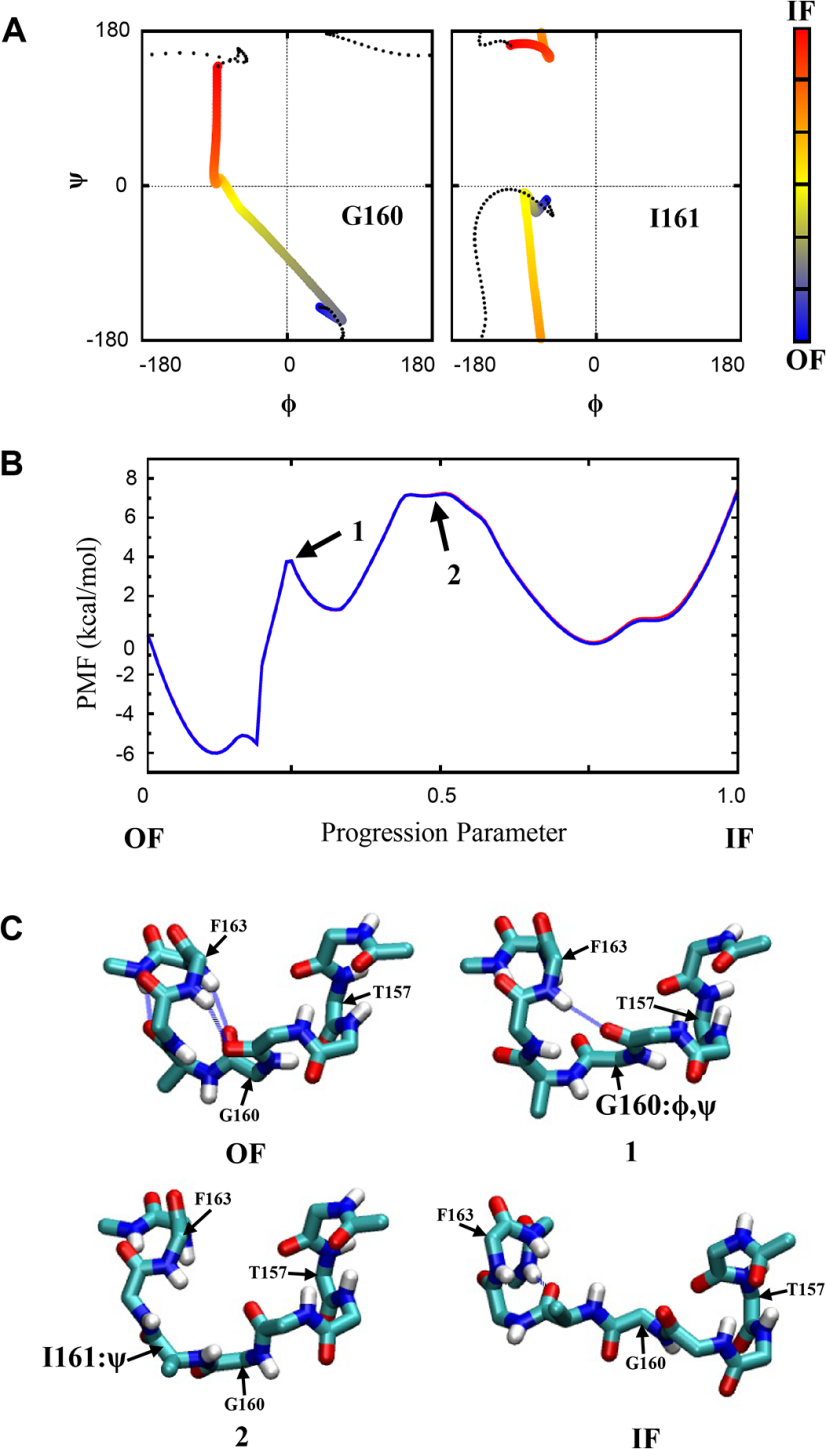
Conformational transition in the isolated IL2 loop. **(A)** The φ and ѱ angles for residues Gly160 and Ile161 along the transition pathway, with the color representing the progression of the transition, from *blue* for the OF state to *red* for the IF state. The *dotted* curves represent the evolution of these torsions in an alternative transition pathway. **(B)** The calculated free energy profile for the transition. The *blue* and *red* curves (which are largely overlapping) were obtained from two independent groups of string-method simulations with different initial coordinates. **(C)** Some snapshots along the transition pathway between the OF and the IF states, including the backbone structures (along with the side chain of Ile161) at the major energetic barriers identified in (B). Major torsion angles involved in each barrier are indicated.

We performed string-method simulations on the isolated EL4 and IL2 loops to refine the observed transitions in the TMD simulations, as described in Methods. Each transition pathway was refined and sampled in two independent groups of string-method simulations with initial coordinates taken from TMD_O→I_ and TMD_I→O_, respectively. Unlike in the intact protein, motions of the isolated loops in these simulations were not restricted by other protein residues. We therefore only performed a small number of refinement iterations for the isolated loops here, to release the strain without introducing too much deviation from the original TMD pathways. The obtained transition pathways with the free energy profiles for the isolated EL4 and IL2 loops are shown in Figs 6 and 7, respectively. For both loops, the calculated free energies from the two groups of simulations with different initial coordinates are almost identical to each other. The free energy profile thus did not appear to depend on the initial coordinates or suffer hysteresis. Because the energetics for the isolated loops (in implicit solvent) and for the loops in the intact protein can be quite different, however, we do not claim that our calculations here properly characterize the conformational thermodynamics of the loops in the real protein environment. Nevertheless, it is clear that the transitions of the loop conformations involve substantial free energy barriers, which is consistent with the behaviors of the unbiased and the TMD simulations discussed earlier for the entire protein.

For the EL4 loop, the two transition pathways identified from TMD_O→I_ and TMD_I→O_ are qualitatively similar. This makes it possible to construct a consensus pathway by averaging the snapshots along the transitions in both simulations, as similarly done in a previous study [54]. Fig 6 displays some properties of the refined transition pathway. Three residues, Gly292, Gly293, and Val294, undergo large changes in their backbone torsion angles during the transition. The projection of the transition pathway onto the φ and ѱ angles of each residue reveals highly nonlinear curves in the Ramachandran plots (Fig 6A). Some features in these plots can be explained by the intrinsic protein backbone structures. For example, when the φ angle of a residue crosses the unfavorable regions around φ = 0°, the ѱ angle is normally about ±90° such that the two adjacent peptide planes are roughly perpendicular to each other to minimize the steric conflicts. From the OF to the IF state, the conformational transition of the EL4 loop involves three subsequent major energetic barriers, as indicated in Fig 6B and 6C. The first barrier is mainly due to a transition in the torsions of Gly292, with the (φ,ѱ) angles changing from ~(75°,45°) in the OF state to ~(-75°,150°) in an intermediate state. The peptide plane between Gly293 and Val294 also undergoes a rotation along with this transition. The second major barrier is associated with a large rotation of the Gly292-Gly293 peptide plane, with the ѱ angle of Gly292 changing from ~150° to ~0°, and the φ angle of Gly293 from ~90° to about -90° as in the IF state. The last energetic barrier is caused by a rotation of the Val294-Ser295 peptide plain, with concerted transitions in the ѱ angle of Val294 and the φ angle of Ser295. After the three major rotations described above, the backbone NH group of Ser295 forms a canonical H-bond with the CO group of Val291, thus wrapping Ser295 as the last turn of the OUT7-8 helix in the IF state.

For the IL2 loop, TMD_O→I_ and TMD_I→O_ exhibit different pathways. In particular, the backbone torsion angles of Gly160 undergo rotations in opposite directions in the two pathways (Fig 7A). Consequently, no consensus pathway can be possibly constructed to represent the two qualitatively different transitions. Therefore, we refined both pathways independently and calculated a free energy profile for each of them. The pathway (from TMD_I→O_) with a lower free energy barrier was then taken as the preferred one. From the OF to the IF state, the conformational transition of the IL2 loop involves two major energetic barriers (Fig 7B and 7C). The first barrier is mainly due to a transition in the torsions of Gly160, with the (φ,ѱ) angles changing from ~(45°,-150°) in the OF state to ~(-60°,-30°) in an intermediate state. In this step, both the H-bond Ile164:H-Gly160:O and the H-bond Arg165:H-Ile161:O break. The second energetic barrier is mainly caused by a large clockwise rotation of the ѱ angle of Ile161, from about -15° in the OF state to ~165° in the IF state. During this rotation, the backbone CO group of Tyr159 loses its H-bond with the backbone NH groups in TM5. When the IF conformation is reached, the CO group of Ile161 forms an H-bond with the NH groups of Ile164 or Arg165 again. The loss of all H-bonds in the middle of the transition may contribute to the high barrier there. Overall, the OF-IF transition in the IL2 loop results in a complete unwrapping of Tyr159 and Gly160 from the N-terminal end of helix TM5.

As mentioned earlier, the initial pathways in the string-method refinement here were taken from the induced transitions in the TMD simulations. The main purpose of the refinement was to relax the loop conformations along the transitions and to obtain a reversible transition pathway for the isolated loops, rather than to significantly alter the initial pathways. The pathways after the refinement (Figs 6 and 7) therefore remain similar to the transitions in the TMD simulations, especially for the EL4 loop where TMD_O→I_ and TMD_I→O_ followed similar pathways. For the IL2 loop where the transitions in the two TMD simulations followed qualitatively different pathways (Fig 7A), the refined pathway here is similar to its initial pathway taken from the transition in the TMD_I→O_ simulation, and different from the transition in TMD_O→I_.

### Loop-driven Simulations

As discussed earlier, the wrapping/unwrapping of the EL4 and IL2 loops may represent some slow degrees of freedom in the conformational transition of the entire protein. To further evaluate the significance of the two loops, we performed two simulations in which external forces were applied to drive the loops from one state to the other, as described in Methods, following the transition pathways determined for the isolated loops. Other parts of the protein were not directly subject to the applied forces here. The purpose of these simulations is to assess the protein structural changes induced by the loop motions.

**Fig 8:**
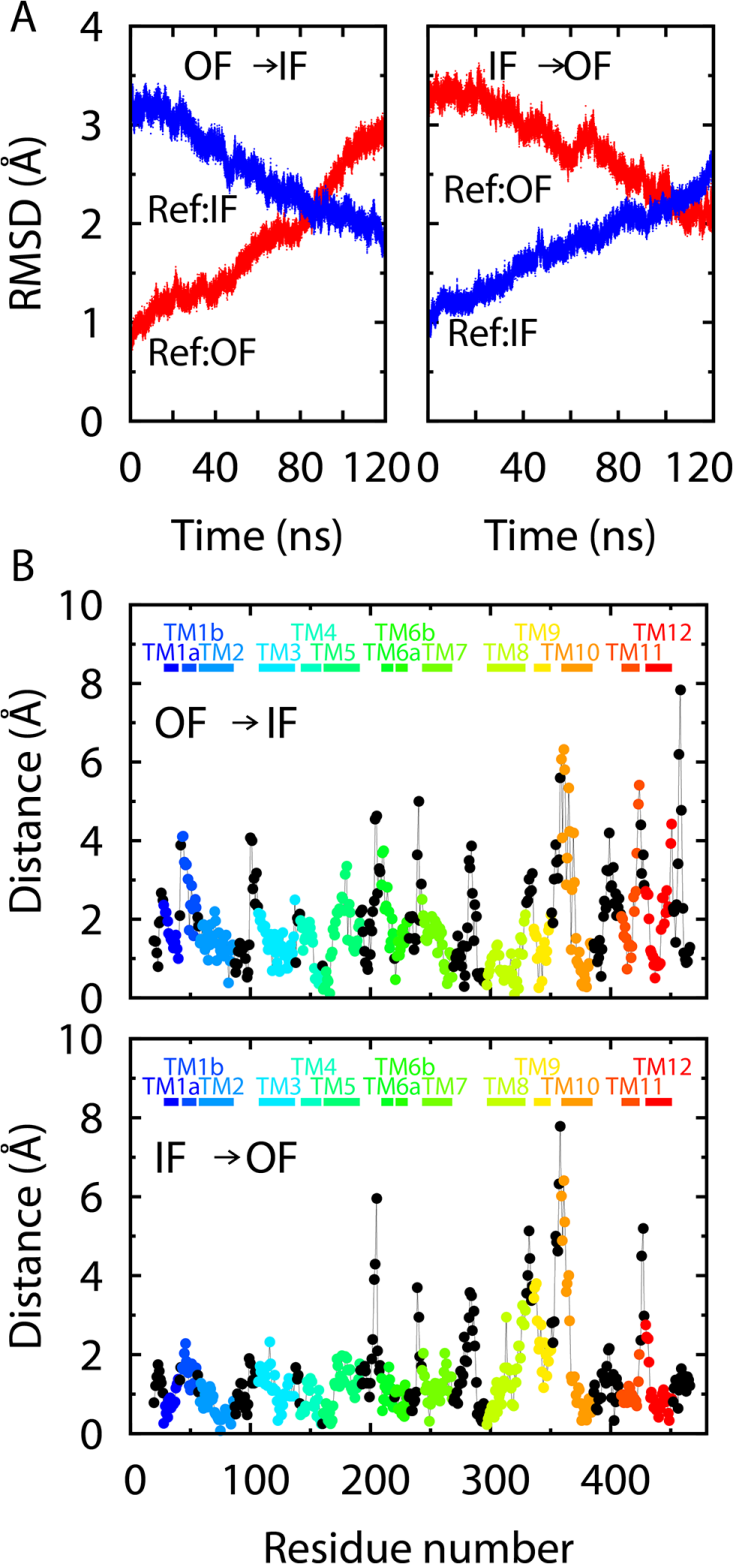
Analysis of the loop-driven simulations. **(A)** RMSDs for the protein C_α_ atoms with respect to the OF and IF crystal structures in the two simulations with the EL4 and IL2 loops driven from the OF to the IF state or from the IF to the OF state. **(B)** A per-residue comparison for the last frames of the loop-driven simulations with respect to the corresponding target crystal structures. After the RMSD alignment, the difference between the C_α_ positions in the end of the loop-driven simulation and in the crystal structure is calculated for each residue and plotted in the figure. The residues in each TM helix are indicated by the bars on the top of the panels.

The trajectories of the two loop-driven simulations showed that a large part of the protein indeed followed the EL4 and IL2 loops, as can be seen from the protein RMSDs to the crystal structures (Fig 8A). In one simulation with the entire protein initially in the OF state, when the two loops were gradually driven to the IF positions, most of the TM helices also approached the IF-state orientations. In the other simulation, similarly, when starting from the IF crystal structure, the protein largely followed the two driven loops toward the OF state. However, some parts of the proteins (Fig 8B), particularly some other inter-helical loops as well as the N-terminal half of TM10 (which forms the extracellular “thin gate” [15]), did not follow the motions of EL4 and IL2 and did not deviate much from their initial positions. Therefore, the two loops alone cannot represent all the slow degrees of freedom in the protein conformation. Nevertheless, unlike EL4 and IL2, other components of the protein have similar secondary structures in the OF and IF states and do not require complicated changes in their local conformations during the transition. Moreover, when the two loops were driven to a (OF or IF) target state, the overall protein structure also became consistent with that state. Our simulations thus demonstrated that the conformational state of the protein can be controlled, to a substantial extent, by the two loops.

## Discussion

In this study, we demonstrated the important roles of the EL4 and IL2 loops in the conformational transition of the ligand-free Mhp1. These loops adopt highly different conformations in the OF and IF structures, which appeared to be largely stable during equilibrium simulations of 100 ns. When the protein structure was driven from one state to the other in the TMD simulations, the loop conformations exhibited resistance and underwent abrupt changes only in the late stages of the simulations. Our free energy calculations on the isolated loops confirmed that the changes of the loop conformations indeed involve nontrivial energetic barriers. Furthermore, when the loops were forced to undergo transitions from one state to the other in our loop-driven simulations, a large part of the protein structure also followed the loops to the target state. In contrast, when the overall structure of the protein was driven (e.g., by restraints on the C_α_ atoms only or on some inter-domain distances) from one state to the other, the loop conformations could not reach the proper target state. Taken together, our simulations suggest that the internal structural changes of the EL4 and IL2 loops would be among the slower degrees of freedom in the conformational transition of the entire protein.

The rocking bundle model [15,58] is a well-accepted mechanism for the conformational change in the LeuT-class transporters including Mhp1. In this model, TMs 3, 4, 8 and 9 of the protein form a “hash motif” and move roughly as a rigid body, and a rotation of the hash motif relative to the other parts of the protein characterizes the conformational transition between the OF and IF states. The EL4 and IL2 loops here serve to connect the hash motif to the protein bundle at the extra- and intracellular sides, respectively. The tilting rotation of the hash motif will increase its distance to the protein bundle at one end while decreasing the distance at the other end. In response, the EL4 and IL2 loops have to adjust their lengths during the conformational transition of the protein. Specifically, from the OF to the IF state, EL4 and IL2 need to become shorter and longer, respectively. Mhp1 appears to control the effective lengths of the loops through their secondary structures. In particular, wrapping some residues to the end of an α-helix will result in a shorter loop length, whereas unwrapping a turn from the α-helix will make the loop longer and more extended. Therefore, our findings here are consistent with the rocking bundle model, and further supplement it with important structural details. Moreover, it also appears plausible that a concerted change in the secondary structures (and thus the effective lengths) of the EL4 and IL2 loops could alter the overall structure of the protein.

Not surprisingly, several of the residues (Gly160, Gly292, Gly293) that undergo large torsional changes during the transition are glycine, which has high conformational flexibility due to the lack of side chain. We expect that mutations of these glycine residues would have substantial effects on the loop conformations, and may alter the structures and equilibrium probabilities for the OF/IF states as well as the transition rates of the protein. We also note that recent distance measurements on Mhp1 [34] and the homologous LeuT transporter [35] indicated that in equilibrium, the OUT7-8 extracellular helix, which is adjacent to the EL4 loop here, may adopt multiple positions relative to other parts of the protein, which might be related to the multiple intermediate metastable states along the EL4 transition pathway identified in this study. Finally, a comparison of the LeuT structures [11] in the OF and IF states also reveals highly different secondary structures in some inter-helical loops, implying that our findings here may be relevant to other membrane transporters as well.

In the calculations of conformational free energies for membrane transporters, the reaction coordinate is often based on holistic measures such as distances between the centers of protein motifs. Ideally, other degrees of freedom orthogonal to the reaction coordinate should be well equilibrated and properly sampled in the simulations. Challenges will arise, however, when some orthogonal degrees of freedom involve slow equilibration, such as slow transitions between two or more metastable states. Because most of the free-energy methods only enhance the sampling along the reaction coordinate(s) but not the orthogonal directions, slow equilibration of the orthogonal coordinates will make the calculated free energy dependent on the initial state of the simulation system, thus giving rise to hysteresis [59]. Even if an accurate free energy as a function of the reaction coordinate can be obtained, it will not reflect the barriers in the orthogonal directions and will likely involve discontinuous changes in the orthogonal coordinates [60], thus not correlating well with the real kinetics of the conformational transition. The wrapping/unwrapping of the inter-helical loops here appears to be such slow degrees of freedom that are not represented by typical reaction coordinates, and would contribute to the hysteresis often encountered in the calculations of protein conformational free energies. Therefore, incorporation of parameters (such as backbone torsions) for the detailed loop conformations into the collective reaction coordinate should improve the convergence and relevance of the free energy calculations. The loop transition pathways identified in this study will help further determine a consistent and reversible pathway for the conformational transition of the entire protein.
